# Spatiotemporal Cluster Detection for COVID-19 Outbreak Surveillance: Descriptive Analysis Study

**DOI:** 10.2196/49871

**Published:** 2024-10-16

**Authors:** Rachel Martonik, Caitlin Oleson, Ellyn Marder

**Affiliations:** 1 Deloitte Arlington, VA United States; 2 Washington State Department of Health Olympia, WA United States

**Keywords:** COVID-19, cluster detection, disease outbreaks, surveillance, SaTScan, space-time surveillance, spatiotemporal, United States, outbreak, outbreaks, pandemic, real-time surveillance, detection, tool, tools, effectiveness, public health, intervention, interventions, community settings, outbreak detection

## Abstract

**Background:**

During the peak of the winter 2020-2021 surge, the number of weekly reported COVID-19 outbreaks in Washington State was 231; the majority occurred in high-priority settings such as workplaces, community settings, and schools. The Washington State Department of Health used automated address matching to identify clusters at health care facilities. No other systematic, statewide outbreak detection methods were in place. This was a gap given the high volume of cases, which delayed investigations and decreased data completeness, potentially leading to undetected outbreaks. We initiated statewide cluster detection using SaTScan, implementing a space-time permutation model to identify COVID-19 clusters for investigation.

**Objective:**

To improve outbreak detection, the Washington State Department of Health initiated a systematic cluster detection model to identify timely and actionable COVID-19 clusters for local health jurisdiction (LHJ) investigation and resource prioritization. This report details the model’s implementation and the assessment of the tool’s effectiveness.

**Methods:**

In total, 6 LHJs participated in a pilot to test model parameters including analysis type, geographic aggregation, cluster radius, and data lag. Parameters were determined through heuristic criteria to detect clusters early when they are smaller, making interventions more feasible. This study reviews all clusters detected after statewide implementation from July 17 to December 17, 2021. The clusters were analyzed by LHJ population and disease incidence. Clusters were compared with reported outbreaks.

**Results:**

A weekly, LHJ-specific retrospective space-time permutation model identified 2874 new clusters during this period. While the weekly analysis included case data from the prior 3 weeks, 58.25% (n=1674) of all clusters identified were timely—having occurred within 1 week of the analysis and early enough for intervention to prevent further transmission. There were 2874 reported outbreaks during this same period. Of those, 363 (12.63%) matched to at least one SaTScan cluster. The most frequent settings among reported and matched outbreaks were schools and youth programs (n=825, 28.71% and n=108, 29.8%), workplaces (n=617, 21.46% and n=56, 15%), and long-term care facilities (n=541, 18.82% and n=99, 27.3%). Settings with the highest percentage of clusters that matched outbreaks were community settings (16/72, 22%) and congregate housing (44/212, 20.8%). The model identified approximately one-third (119/363, 32.8%) of matched outbreaks before cases were associated with the outbreak event in our surveillance system.

**Conclusions:**

Our goal was to routinely and systematically identify timely and actionable COVID-19 clusters statewide. Regardless of population or incidence, the model identified reasonably sized, timely clusters statewide, meeting the objective. Among some high-priority settings subject to public health interventions throughout the pandemic, such as schools and community settings, the model identified clusters that were matched to reported outbreaks. In workplaces, another high-priority setting, results suggest the model might be able to identify outbreaks sooner than existing outbreak detection methods.

## Introduction

By March 2021, a total of 340,323 COVID-19 cases were reported in Washington State. During the peak of the winter 2020-2021 surge, the weekly incidence rate was 284.9 per 100,000 population and the number of reported outbreaks per week was 231 (Washington Disease Reporting System, unpublished data, 2023). It is important to identify and investigate outbreaks of COVID-19 to reduce the spread of the disease and protect vulnerable populations, such as long-term care facility residents [1,2]. Outbreaks in these settings are a high priority because residents have a greater risk of severe outcomes [3]. However, case and outbreak investigations can be resource-intensive [4]; thus, it is helpful to use automated and systematic methods as much as possible. To identify outbreaks in long-term care facilities, the Washington State Department of Health (WA DOH) routinely identified cases occurring at known health care facility addresses through an automated process; results are shared with local health jurisdictions (LHJs) [5].

In Washington State, there are 35 LHJs, which are the administrative public health authorities that manage and oversee health services at the county or regional level, including case and outbreak investigations for reportable diseases such as COVID-19. Aside from facility address matching, other systematic methods for cluster detection were rare. Reported outbreaks likely undercounted the true number of outbreaks due to varying detection methods and data disruptions. When the volume of cases dramatically increased during short periods of time, health care systems, including hospitals, laboratories, and public health, experienced delays which impacted the completeness and timeliness of reporting and processing case information. Furthermore, limited testing capacity during surges resulted in underreporting of mild or asymptomatic cases, further contributing to incomplete data [6-10]. To aid outbreak detection using systematic and automated methods, WA DOH initiated statewide cluster detection using SaTScan, implementing a space-time permutation model to identify timely and actionable COVID-19 clusters for local investigation.

Infectious disease space-time surveillance allows health officials to target resources and interventions in specific areas of emerging disease [11,12]. Scan statistics are a public health surveillance method using data models to identify and evaluate emerging clusters of cases in a temporal, spatial, or space-time setting [13]. SaTScan is a free software widely used for spatial-temporal analysis. In public health, SaTScan has been used for many reportable conditions [14], including foodborne illnesses [15-17], Lyme disease [18], HIV [19], and opioid overdoses [20,21]. Space-time cluster detection of COVID-19 cases was implemented throughout the pandemic in many jurisdictions [11,22-26]. SaTScan is a flexible and adaptable tool, allowing users to specify a variety of parameters (eg, analysis type, probability model type, geographic and temporal data aggregation, study period length, or spatial and temporal cluster sizes) [26]. The user can tailor the analysis to their specific data sets and objectives through this customization. Daily prospective Poisson models [11,22,23,27] were frequently used for COVID-19; however, models varied based on data availability, the geographical area of study, and specific study goals [12,28]. This report details our efforts to implement a customized systematic approach for COVID-19 cluster detection in Washington State using SaTScan along with an assessment of the tool’s effectiveness.

## Methods

### Data Sources and Model Development

#### Case Data

During this period, laboratories in Washington State were required to report all SARS-CoV-2 test results to public health. The results included specimen collection date and patient demographics. COVID-19 cases were defined according to national surveillance [29]. Cases were geocoded based on residential address using WA DOH’s Geocoder Web Service. Cases were excluded if the patient resided out of state, did not have a positive molecular or antigen test during the model study period (which is defined as the time period included in each analysis), or if their address could not be geocoded.

#### Model Development

For the pilot, we chose a state-level retrospective space-time permutation model. The space-time permutation model was used because it does not require population testing rates which were not reliably available at the time. Case patients were aggregated on specimen collection date, as a proxy for infection date, and census tract of their residential address, which was the most complete address available. We selected a maximum cluster duration of 7 days based on the median incubation period of COVID-19 [30]. Given this maximum cluster duration, we selected 21 days for this study’s period, which is the minimum recommended (ie, 3 times the maximum cluster duration). We used a day-of-week adjustment given the variability in reporting on weekends. We tested 3 maximum cluster radii of 5 km, 10 km, and 20 km to determine which provided the best yield across urban and rural settings. We used a 3-day lag to account for data reporting delays.

In total, 6 LHJs (Benton-Franklin Health District, Clark County Public Health, Kitsap Public Health District, Tacoma-Pierce County Health Department, Spokane Regional Health District, and Whatcom County Health Department) participated in the pilot and provided feedback on the initial model. LHJs reported that the clusters identified from the initial model were larger (in both size and radius) than were actionable. Based on this feedback, we tested the following parameters to identify smaller, more actionable clusters: for analysis type, statewide versus LHJ-specific and weekly retrospective versus daily prospective; for geographic aggregation, census tract versus census block group; for maximum cluster radius, 5 km versus 10 km versus 20 km; and for data lag, 1-day versus 2-day versus 3-day. To compare the retrospective and prospective models, we ran the daily model for 14 days and the weekly model on days 7 and 14. To determine the most appropriate lag, we analyzed data over several weeks using each lag and compared the identified clusters. We used heuristic criteria, prioritizing smaller (size and radius) and timely clusters, to finalize the parameters and features. Timely clusters were defined as having occurred within 1 week of the analysis—early enough for intervention to prevent further transmission. The final model was an LHJ-specific, retrospective space-time permutation with case patients aggregated on specimen collection date and census block group of their residential address. The maximum cluster duration was 7 days, the study period was 21 days, and the maximum cluster radius was 20 km. We used a day-of-week adjustment and a 2-day lag.

#### SaTScan Cluster Data

The SaTScan cluster data set included all clusters detected since the inaugural weekly analysis in August through December 2021, representing cases with specimen collection dates from July 17 to December 17, 2021. This period was selected for its relatively stable incidence and predominance of the Delta variant. Variables included were LHJ, cluster start and end date, radius, number of cases, and *P* value. Statistical significance was defined as α<.1 to account for multiple testing. Due to the weekly retrospective design with the 21-day study period, the same or similar cluster could be identified in subsequent weeks. To deduplicate clusters included in this comparison, we compared clusters week-to-week using the Jaccard similarity (JS) index [31]. Clusters with a JS index ≤12% were categorized as new; all other clusters were considered ongoing.

#### Reported Outbreak Data

Outbreak detection and reporting varies statewide and by outbreak setting. Known COVID-19 outbreaks are captured in our state surveillance system, where they are linked to cases. Due to COVID-19 outbreak definitions and reporting requirements differing by setting and changing over time, we applied a standardized definition for this analysis. Outbreaks were defined as ≥2 cases that were associated with an outbreak event in the state surveillance system and had specimen collection dates within 14 days of each other. Start and end dates were based on the first and last specimen collection date. We included outbreaks with start and end dates from July 17 to December 17, 2021, and collapsed the original 64 outbreak settings into 11 broad categories: school and youth programs, workplace, retail, congregate housing, food and beverage establishments, community, colleges, travel, long term care facilities, and outpatient and inpatient settings. Outbreaks linked to military settings as indicated by LHJs were excluded.

### Analysis

#### Descriptive Analysis

We compared SaTScan clusters among LHJs categorized by population size. LHJs with a population ≥400,000 persons were classified as large, 90,000-399,999 as medium, 25,000-89,999 as small, and ≤24,999 as rural. Statistical significance was determined using chi-square and Wilcoxon tests, using α<.05. To assess correlation among study period incidence (SPI) and cluster characteristics, we calculated Pearson correlation coefficients. SPI was defined as the average number of cases per 100,000 population during the SaTScan 21-day study period. Cluster size was defined as the number of cases per cluster and timeliness as clusters ending within 1 week before the analysis date.

#### Outbreak-Cluster Comparison

To assess the ability of the model to detect true outbreaks, we matched SaTScan clusters, including statistically significant and nonsignificant clusters, with reported outbreaks. We validated a random sample of outbreak-cluster pairs with ≥1 case in common to refine the matching criteria. Due to the mean size of outbreaks and clusters vastly differing, we selected 2 matching criteria. First, ≥20% of outbreak cases had to be identified in a cluster and, second, ≥6% of cluster cases had to be identified in an outbreak. We calculated summary statistics of the matched pairs. Matched and unmatched outbreaks were compared to determine the types and characteristics of outbreaks identified as SaTScan clusters. Statistical significance was assessed using chi-square tests and α<.05. We used R (R Core Team, 2021) to run SaTScan (version 9.6) and for all other analyses.

### Ethical Considerations

Original data collection was conducted as part of WA DOH COVID-19 surveillance. As such, per the Office for Human Research Protections guidelines, institutional review board approval was not required [32]. As part of public health surveillance activities authorized by a public health authority, the secondary analysis of the data described in this study was excluded from the definition of research provided by the revised Common Rule [33] and did not receive approval or an exemption from an institutional review board. The researchers were granted access to the data as part of their official duties within the WA DOH.

## Results

### Model Development

During pilot testing, the statewide model identified clusters that were large in radius and size, which did not meet the goal of actionable clusters. Independent models for each LHJ with the same parameters produced smaller clusters, which did meet that goal. Therefore, our final design included LHJ-specific models that were combined to provide statewide results.

Next, we compared daily prospective and weekly retrospective models. There were 12 significant clusters identified by the daily prospective model; 10 (83%) of which were also identified by the weekly retrospective model. While the daily prospective used more recent data, the model required more resources to produce and review. Given the overlap between the model output and resource constraints, we selected the weekly retrospective analysis for the final model.

In comparing 1-, 2-, and 3-day lag periods, we found about half (47/90, 52%) identified similar clusters regardless of lag. The remainder identified similar clusters across the 2- and 3-day lag. We chose a 2-day lag to maximize timeliness.

The initial model, which aggregated cases to census tracts, produced clusters that were too large geographically for public health action. Using the smaller unit of census block group was favorable based on heuristic criteria.

Lastly, we tested various maximum cluster radii. The 5-km model identified the smallest clusters (median radius 3.3, range 0-5, IQR 1.5-4.5 km), followed by the 10-km model (median radius 5.2, range 0-10, IQR 2.3-8.3 km), and then the 20-km model (median radius 6.1, range 0-19.5, IQR 2.9-11.3 km). The 20-km radius was determined to capture both smaller clusters in urban areas and larger clusters in rural areas, and thus was selected for the final model.

### Descriptive Analysis

From July 17 to December 17, 2021, there were 341,505 COVID-19 cases reported among Washington State residents. Of these, 316,642 (92.72%) had a geocoded residential address. A median of 41,217 cases was included in each weekly SaTScan analysis (range 24,996-61,069, IQR 30,863-52,842); a total of 4659 clusters were identified (weekly median 236, range 197-275, IQR 218-248). Clusters included 98,172 unique cases (28.74% of reported COVID-19 cases); most (2874/4659, 61.68%) were new clusters. The remaining analyses are limited to new clusters.

Among the 2874 new clusters, 887 (30.86%) occurred in large LHJs, 890 (30.96%) in medium LHJs, 734 (25.54%) in small LHJs, and 363 (12.63%) in rural LHJs (Tables 1 and 2). Overall, the median cluster size was 15 cases (range 2-1045, IQR 8-29) and significantly differed across population groups (large *P<*.001, medium *P*<.001, small *P<*.001, and rural *P<*.001). Rural LHJs had the smallest median cluster size (4 cases, range 2-28, IQR 3-6) and large LHJs the largest (27 cases, range 5-1045, IQR 16-49). The median number of weekly clusters per LHJ also differed significantly: rural LHJs had the least (2, range 1-6, IQR 1-3; *P<*.001), followed by small (3, range 1-9, IQR 2-4; *P<*.001), medium (5, range 1-12, IQR 3-6; *P*<.001), and large LHJs (9, range 2-21, IQR 6-11; *P<*.001). Overall, the median cluster radius was 2.4 (IQR 0.9-6.88) km. The radius significantly differed for medium (2.5 km; *P=*.045), small (3.8 km; *P<*.001), and rural LHJs (0 km; *P*<.001). Of the 2874 clusters, 486 (16.91%) were statistically significant in the weekly analysis. Rural LHJs had a significantly smaller percentage of significant clusters (37/363, 10.2%; *P<*.001). There was no significant difference in duration (overall 4 days, range 1-7) or timeliness (overall 1674/2874, 58.24%) by the LHJ population group.

**Table 1:** Characteristics of new COVID-19 clusters identified by the SaTScan model, by LHJ^a^ population group, Washington State, July 17 to December 17, 2021.

	LHJ population group^b^												Total (N=2874), median (IQR, range)
	Large (n=887)		Medium (n=890)			Small (n=734)			Rural (n=363)				
	Median (IQR, range)	*P* value	Median (IQR, range)	*P* value	Median (IQR, range)		*P* value	Median (IQR, range)		*P* value			
Cluster size^c,d^	27 (16-49, 5-1045)	<.001	17 (10-30, 2-362)	<.001	9 (5-15, 2-113)		<.001	4 (3-8, 2-28)		<.001	15 (8-29, 2-1045)		
Weekly clusters per LHJ^d^	9 (6-11, 2-21)	<.001	5 (3-6, 1-12)	<.001	3 (2-4, 1-9)		<.001	2 (1-3, 1-6)		<.001	4 (2-6, 1-21)		
Radius (km)^d^	2.2 (1.3-4.2, 0-19.9)	.30	2.5 (1.2-6.3, 0-20)	.045	3.8 (0-11.8, 0-20)		<.001	0 (0-7.7, 0-19.8)		<.001	2.4 (0.9-6.8, 0-20)		
Duration (days)^d^	4 (3-6, 1-7)	<.001	4 (2-6, 1-7)	.11	4 (2-6, 1-7)		<.001	4 (2-5, 1-7)		.002	4 (2-6, 1-7)		

^a^LHJ: local health jurisdiction.

^b^Large county population ≥400,000 persons, medium 90,000-399,999 persons, small 25,000-89,999 persons, and rural ≤24,999 persons.

^c^Cases per cluster.

^d^Statistical significance tested with unpaired 2-sided Wilcoxon test.

**Figure 1:**
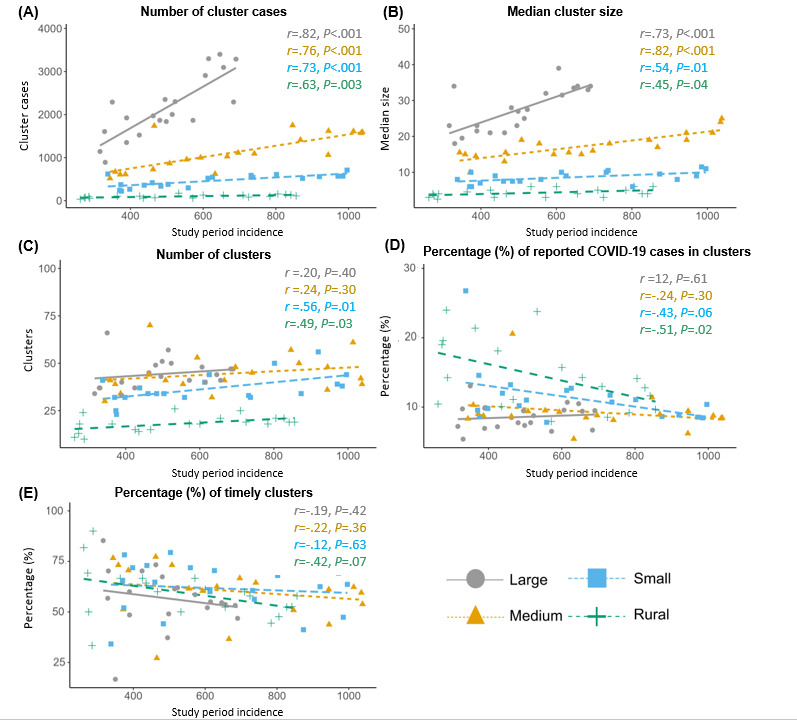
Study period incidence by cluster characteristics stratified by LHJ population group. LHJ: local health jurisdiction.

The SPI was significantly positively correlated with the weekly number of cluster cases among large (*r*=.82; *P*<.001), medium (*r*=0.76; *P=*.01), and small (*r*=0.73; *P=*.01) LHJs and with cluster size among large (*r*=0.73; *P=*.01) and medium LHJs (*r*=0.82; *P*<.001; Figure 1). There were few nonsignificant moderate correlations between SPI and median cluster size, number of weekly clusters, and percentage of reported cases associated with clusters. There was no correlation between the timeliness of clusters and SPI (large *r*=–0.19, medium *r*=–0.22, small *r*=–0.12, and rural *r*=–0.42).

**Table 2:** Characteristics of new COVID-19 clusters identified by the SaTScan model, by LHJ^a^ population group, Washington State, July 17 to December 17, 2021.

	LHJ population group^b^												Total (N=2874), n (%)
	Large (n=887)		Medium (n=890)			Small (n=734)			Rural (n=363)				
	n (%)	*P* value	n (%)	*P* value	n (%)		*P* value	n (%)		*P* value			
Significant clusters^c,d^	162 (18.3)	.30	171 (19.2)	.47	116 (15.8)		.07	37 (10.2)		.002	486 (16.91)		
Timely clusters^c,e^	490 (55.2)	.27	525 (59)	.81	452 (61.6)		.29	207 (57)		.83	1674 (58.24)		

^a^LHJ: local health jurisdiction.

^b^Large county population ≥400,000 persons, medium 90,000-399,999 persons, small 25,000-89,999 persons, and rural ≤24,999 persons.

^c^Statistical significance tested with chi-square test.

^d^Cluster’s *P* value is <.01.

^e^Cluster’s end date was within 1 week of analysis date.

### Outbreak-Cluster Comparison

There were 2874 reported outbreaks from July 17 to December 17, 2021, and 363 (12.63%) matched to ≥1 SaTScan cluster (Table 3). Among reported outbreaks, the most common settings were schools and youth programs (825/2874, 28.71%), workplaces (617/2874, 21.47%), and long-term care facilities (541/2874, 18.82%). Among the 363 matched outbreaks, the most common settings were also schools and youth programs (108/363, 29.8%), long-term care facilities (99/363, 27.3%), and workplaces (56/363, 15.4%), as well as congregate housing (44/363, 12.1%). Of the 2874 reported outbreaks, the settings with the largest percentage of reported outbreaks that matched clusters were community settings (16/72, 22%), congregate housing (44/212, 20.8%), long-term care facilities (99/541, 18.3%), and school and youth programs (108/825, 13.1%). Settings with <10% of matched outbreaks were workplaces (56/617, 9.1%), food and beverage establishments (10/113, 8.8%), inpatient (6/77, 8%), outpatient (8/104, 7.7%), colleges (1/16, 6%), and retail settings (14/287, 4.9%).

**Table 3:** Reported outbreaks compared with matched^a^ outbreaks by setting type, Washington State, July 17 to December 17, 2021.

		Reported outbreaks, n (%)	Matched outbreaks					
			N	Column percentage, %	Row percentage, %	*P* value (chi-square test)	Report date after analysis date^b^, n/n (%)	*P* value (chi-square test)
**Nonhealth care settings**								
	Schools and youth programs	825 (28.71)	108	29.8	13.1	.63	42/108 (38.9)	<.001
	Workplace^c^	617 (21.47)	56	15.4	9.1	.003	24/56 (42.9)	<.001
	Retail settings^d^	287 (9.99)	14	3.9	4.9	<.001	7/14 (50)	.16
	Congregate housing^e^	212 (7.38)	44	12.1	20.8	<.001	8/44 (18.2)	<.001
	Food and beverage establishments^f^	113 (3.93)	10	2.8	8.8	.22	6/10 (60)	.62
	Community^g^	72 (2.5)	16	4.4	22	.01	4/16 (25)	<.001
	Colleges	16 (0.6)	1	0.3	6	.44	1/1 (100)	—^h^
	Travel	10 (0.3)	1	0.3	10	.80	1/1 (100)	—
**Health care settings**								
	Long-term care facilities^i^	541 (18.82)	99	27.3	18.3	<.001	20/99 (20)	<.001
	Outpatient	104 (3.62)	8	2.2	7.7	.12	5/8 (63)	.77
	Inpatient	77 (2.7)	6	1.7	8	.20	1/6 (17)	.24
	Total	2874 (100)	363	100	12.63	—	119/363 (32.8)	—

^a^Matched is defined as ≥20% of outbreak cases identified in a SaTScan cluster and ≥6% of SaTScan cluster cases identified in an outbreak.

^b^All cases were associated with the outbreak event in the surveillance system after the SaTScan cluster was identified.

^c^Workplace manufacturing (food: n=24; nonfood: n=162), agricultural employee provided housing (n=71), construction (n=49), professional services or office-based (n=43), utilities (n=38), agencies, facilities, and similar (n=37), and other settings (n=193).

^d^Retail services includes grocery (n=144), retail (n=129), personal care and service (hair or nails; n=13), and other (n=1).

^e^Congregate housing includes shelter or homeless services (n=126), correctional settings (n=59), congregate housing (n=10), juvenile justice settings (n=6), and others (n=11).

^f^Food and beverage establishments include food service or restaurants (n=101), bars or nightclubs (n=11), and others (n=1).

^g^Community includes those in places of worship (n=27), hospitality (n=16), large gatherings (n=13), private events (n=11), and others (n=16).

^h^Not available.

^i^Long-term care facilities include assisted living (n=260), nursing homes (n=158), adult family homes (n=88), enhanced services facilities or intermediate care facilities for individuals with intellectual disabilities (n=18), senior living (n=14), and others (n=3).

The median duration among all reported outbreaks was 8 days (range 1-151, IQR 4-14) and 10 days (range 1-151, IQR 5-16) among matched outbreaks. Among all reported outbreaks, the median size was 4 cases (range 2-256, IQR 2-7) and among matched outbreaks, it was 6 cases (range 2-232, IQR 3-14).

Outbreaks in community settings (16/72, 22%; *P*=.01), congregate housing (44/212, 20.8%; *P*<.001), and long-term care facilities (99/541, 18.3%; *P*<.001) were more likely to match to clusters than other settings, while outbreaks in workplaces (56/617, 9.1%; *P*=.003) and retail settings (14/287, 4.9%; *P*<.001) were less likely to match. For one-third (119/363, 32.8%) of matched outbreaks, all cases were associated with the outbreak event in the surveillance system after the SaTScan cluster was identified. This resulted in a “report date” later than the “analysis date.” Of the 363 matched outbreaks, those in workplaces (24/56, 43%; *P*<.001) and schools and youth program settings (42/108, 38.9%; *P*<.001) were more likely to have report dates later than analysis dates, while outbreaks in community settings (4/16, 25%; *P*<.001), long-term care facilities (20/99, 20%; *P*<.001), and congregate housing (8/44, 18.2%; *P*<.001) were less likely (Table 3).

A reported outbreak could match with more than one cluster and vice versa. The 363 outbreaks matched 349 SaTScan clusters, which resulted in 384 unique outbreak-cluster pairs. Matched outbreaks had a median size of 6 (IQR 3-14) cases and a duration of 10 (IQR 5-16) days. Matched clusters had a median size of 17 (IQR 12-34) cases and a duration of 5 (IQR 3-6) days. The median number of linked cases (those common to the outbreak and cluster in a matched pair) was 3 (range 1-58, IQR 1-6). This represents a median of 40% (IQR 26.4%-60%) of outbreak cases that were part of the cluster and 12.5% (IQR 8.3%-25%) of cluster cases that were part of the outbreak. The median JS was 10% (IQR 7.2%-17.8%; Tables 4 and 5). For an illustrative example, an 8-case workplace outbreak matched a 29-case cluster by 8 linked cases, with 100% (8/8) of outbreak cases in the cluster and 28% (8/29) of cluster cases in the outbreak, resulting in JS was 28%.

**Table 4:** Characteristics of COVID-19 reported outbreaks and detected clusters that matched, Washington State, July 17 to December 17, 2021^a^.

	Matched outbreaks^a^ (n=363), median (IQR, range)	Matched clusters (n=349), median (IQR, range)
Number of cases	6 (3-14, 2-232)	17 (12-34, 4-147)
Duration (days)	10 (5-16, 1-151)	5 (3-6, 1-7)

^a^Matched is defined as ≥20% of outbreak cases identified in a SaTScan cluster and ≥6% of cluster cases identified in an outbreak.

**Table 5:** Characteristics of COVID-19 reported outbreaks and detected clusters that matched, Washington State, July 17 to December 17, 2021^a^.

	Matched outbreak-cluster pairs (n=384), median (IQR, range)
Number of linked cases	3 (1-6, 1-58)
Outbreak cases linked (%)	40 (26.4-60, 20-100)
Cluster cases linked (%)	12.5 (8.3-25, 6-100)
Jaccard similarity (%)	10 (7.2-17.8, 5-100)

^a^Matched is defined as ≥20% of outbreak cases identified in a SaTScan cluster and ≥6% of cluster cases identified in an outbreak.

## Discussion

### Principal Findings

During a time when LHJs were heavily burdened with case and outbreak investigations, WA DOH initiated systematic statewide cluster detection for COVID-19 using SaTScan. SaTScan parameters can be adjusted based on study objectives, data availability, and disease and population characteristics [26,34]. Washington State required a model tailored to its overtaxed information systems and decentralized public health system, which serves almost 8 million residents in 39 diverse counties. We collaborated with LHJs to implement a space-time permutation model that maximized real-time surveillance data and identified opportunities for public health intervention.

We assessed a daily prospective model, which would have provided the most real-time surveillance; however, it did not produce substantially different results than a weekly retrospective model. Considering the time and resource constraints faced by LHJs, we determined the weekly retrospective model was the most appropriate. Despite having parameters unique from other studies [11,22,23], the model worked well in Washington and identified clusters early enough for intervention. It is important to identify outbreaks as quickly as possible (when there are fewer cases) to prevent further transmission and illness. While outbreak size may vary due to population and disease transmission levels, our analysis showed that 58.24% (1674/2874) of clusters were timely regardless of population or incidence. By identifying clusters promptly, LHJs can conduct targeted investigations, implement containment measures quickly, and prioritize resource distribution to areas with the greatest need.

The number of weekly clusters and cluster size were both correlated with population; more and larger clusters were identified in highly populated urban areas which generally have larger health departments with greater epidemiologic capacity [35]. These data support the use of a systematic statewide approach as it benefits LHJs with varying populations and resource capacity, ensuring that all LHJs, regardless of resources, can effectively manage outbreaks by leveraging a standardized, statewide surveillance system.

Space-time scan statistics have successfully identified respiratory disease outbreaks [13,23,36], including COVID-19 [37]. To determine if the model could identify true outbreaks, we compared clusters with reported outbreaks. Generally, we did not expect a high degree of alignment between reported outbreaks and SaTScan clusters, given SaTScan was intended to fill gaps in current outbreak detection methods. Given the use of residential address as the geographic input for the model, outbreaks in congregate living settings (eg, long-term care facilities or corrections) had a high cluster matching rate as expected. Outbreaks that occur further from the home, such as in workplaces, would likely be harder for the model to detect.

During the COVID-19 pandemic, several settings have been a high priority and focus for public health interventions, including workplaces, community settings, and schools. Workplaces, the second most common setting among reported outbreaks in Washington State, play a central role in the COVID-19 community transmission [38-40] and have been subject to safety prevention requirements throughout the pandemic [41] to reduce the spread of the disease. As expected, workplace outbreaks matched fewer clusters than all reported outbreaks. Incorporating workplace addresses may have helped, however occupational and industry data for COVID-19 are largely incomplete and often not standardized, which may create barriers to systematically identifying workplace outbreaks [39,42]. Efforts to collect and standardize these data and improvement of occupational health surveillance may address this [38].

Community settings have also been a high priority for public health interventions. In fall 2021, when SaTScan was first implemented in Washington State, many efforts were made to implement safety precautions, including limiting capacity at events and gatherings [43], masking [44], and vaccination requirements at large events [45]. Outbreaks among community settings made up only 2.51% (72/2874) of all reported outbreaks, yet 22.2% (16/72) of these outbreaks were linked to clusters, suggesting SaTScan might be a useful method for systematically detecting these types of outbreaks.

The model performed moderately well for schools and youth programs, another high priority and focus of public health interventions. For the 2021-2022 school year in Washington State, public health officials collaborated on guidance to allow students to return safely to in-person instruction [46]. Given their residential proximity, we expected a high cluster match rate for educational settings. Schools and youth programs were the most common setting among reported outbreaks as well as matched outbreaks. However, only 13.1% (108/825) of school outbreaks matched a cluster compared to 12.63% (363/2874) overall, arguably leaving room for improvement. The model could be further tailed to capture outbreaks in educational settings by incorporating school district-level models or including age as an input variable.

Lastly, about a third of outbreaks that matched clusters had a report date in the surveillance system that was later than when the cluster was identified by the model. This suggested that the model might be able to identify outbreaks earlier than existing outbreak detection methods. Detecting outbreaks sooner allows for quicker implementation of containment measures, reducing the spread of the disease and minimizing the impact on communities. By automating the detection process, local public health workers can spend more time on the outbreak intervention measures and less on the initial outbreak detection and investigation work.

The findings in this report are subject to several limitations. First, the model does not account for irregular geographics, such as major waterways. Second, residential address was used to determine outbreak detection as this was the most complete and available. However, residential addresses may not be the most representative of where exposure occurs and may limit the identification of clusters where transmissions happened away from home. Third, reported outbreaks are identified through many indicators and sources that vary by LHJ, contain known biases including lags or gaps during surges [6-9], and likely underestimate actual outbreaks [47,48]. Fourth, because the final model was run independently for each LHJ, cross-jurisdictional clusters were not identified and thus not matched with similar outbreaks. Lastly, as expected, relatively few SaTScan clusters matched outbreaks, and without further investigation to identify epidemiologic or genomic linkages, we cannot determine if unmatched clusters are true outbreaks.

### Conclusion

Our goal was to implement a statewide systematic cluster detection process to identify COVID-19 clusters. WA DOH successfully developed and implemented a SaTScan space-time permutation model that met these goals within the state’s unique structure and systems. Regardless of the LHJ population, the model identified reasonably sized, timely clusters for investigation and resource prioritization. Cluster size increased with incidence but likely remained actionable even for smaller LHJs. Among reported outbreaks that matched clusters, the model performed well in congregate living settings, schools, and events, which have been high-priority settings throughout the COVID-19 pandemic. There is an opportunity to tailor the model to further improve cluster detection in some settings, such as workplaces and schools. Evidence suggests running a weekly model might identify some outbreaks sooner than existing outbreak detection methods, particularly in workplace outbreaks where data collection remains a challenge. In summary, our SaTScan model was able to identify timely, actionable clusters, especially in high-priority settings, which can serve to support outbreak detection to reduce further COVID-19 transmission.

## Abbreviations

JS: Jaccard similarity
LHJ: local health jurisdiction
SPI: study period incidence
WA DOH: Washington State Department of Health

## Data Availability

The data sets generated and analyzed during our study are not publicly available due to the presence of personally identifiable information. For privacy reasons, these data will not be made available upon request.

## References

[1] McMichael TM, Currie DW, Clark S, Pogosjans S, Kay M, Schwartz NG, Lewis J, Baer A, Kawakami V, Lukoff MD, Ferro J, Brostrom-Smith C, Rea TD, Sayre MR, Riedo FX, Russell D, Hiatt B, Montgomery P, Rao AK, Chow EJ, Tobolowsky F, Hughes MJ, Bardossy AC, Oakley LP, Jacobs JR, Stone ND, Reddy SC, Jernigan JA, Honein MA, Clark TA, Duchin JS (2020). Epidemiology of Covid-19 in a Long-Term Care Facility in King County, Washington. N Engl J Med.

[2] Pray IW, Kocharian A, Mason J, Westergaard R, Meiman J (2021). Trends in outbreak-associated cases of COVID-19—Wisconsin, March-November 2020. MMWR Morb Mortal Wkly Rep.

[3] Telford CT, Onwubiko U, Holland DP, Turner K, Prieto J, Smith S, Yoon J, Brown W, Chamberlain A, Gandhi N, Williams S, Khan F, Shah S (2020). Preventing COVID-19 outbreaks in long-term care facilities through preemptive testing of residents and staff members—Fulton County, Georgia, March-May 2020. MMWR Morb Mortal Wkly Rep.

[4] Bonney T, Grant MP (2023). Local health department engagement with workplaces during the COVID-19 pandemic-examining barriers of and facilitators to outbreak investigation and mitigation. Front Public Health.

[5] Wu C, Tasslimi A (2024). Enhanced COVID-19 surveillance methods for early detection in high-risk settings: Utilizing fuzzy address matching and exact phone number matching to improve case identification in congregate care and correctional facilities.

[6] Pagel C, Yates CA (2021). Tackling the pandemic with (biased) data. Science.

[7] Britton T, Scalia Tomba G (2019). Estimation in emerging epidemics: biases and remedies. J R Soc Interface.

[8] Overton CE, Stage HB, Ahmad S, Curran-Sebastian J, Dark P, Das R, Fearon E, Felton T, Fyles M, Gent N, Hall I, House T, Lewkowicz H, Pang X, Pellis L, Sawko R, Ustianowski A, Vekaria B, Webb L (2020). Using statistics and mathematical modelling to understand infectious disease outbreaks: COVID-19 as an example. Infect Dis Model.

[9] Boore A, Herman KM, Perez AS (2010). Surveillance for foodborne disease outbreaks—United States, 2007. MMWR.

[10] Badker R, Miller K, Pardee C, Oppenheim B, Stephenson N, Ash B, Philippsen T, Ngoon C, Savage P, Lam C, Madhav N (2021). Challenges in reported COVID-19 data: best practices and recommendations for future epidemics. BMJ Glob Health.

[11] Hohl A, Delmelle EM, Desjardins MR, Lan Y (2020). Daily surveillance of COVID-19 using the prospective space-time scan statistic in the United States. Spat Spatiotemporal Epidemiol.

[12] Leal-Neto OB, Santos FAS, Lee JY, Albuquerque JO, Souza WV (2020). Prioritizing COVID-19 tests based on participatory surveillance and spatial scanning. Int J Med Inform.

[13] Kulldorff M, Heffernan R, Hartman J, Assunção R, Mostashari F (2005). A space-time permutation scan statistic for disease outbreak detection. PLoS Med.

[14] Greene SK, Peterson ER, Kapell D, Fine AD, Kulldorff M (2016). Daily reportable disease spatiotemporal cluster detection, New York City, New York, USA, 2014-2015. Emerg Infect Dis.

[15] Hughes G J, Gorton R (2013). An evaluation of SaTScan for the prospective detection of space-time campylobacter clusters in the North East of England. Epidemiol Infect.

[16] Edens C, Alden NB, Danila RN, Fill MA, Gacek P, Muse A, Parker E, Poissant T, Ryan PA, Smelser C, Tobin-D'Angelo M, Schrag SJ (2019). Multistate analysis of prospective Legionnaires' disease cluster detection using SaTScan, 2011-2015. PLoS One.

[17] Latash J, Greene SK, Stavinsky F, Li S, McConnell JA, Novak J, Rozza T, Wu J, Omoregie E, Li L, Peterson ER, Gutelius B, Reddy V (2020). Salmonellosis outbreak detected by automated spatiotemporal analysis—New York City, May-June 2019. MMWR Morb Mortal Wkly Rep.

[18] Kugeler KJ, Farley GM, Forrester JD, Mead PS (2015). Geographic distribution and expansion of human lyme disease, United States. Emerg Infect Dis.

[19] Hixson BA, Omer SB, del Rio C, Frew PM (2011). Spatial clustering of HIV prevalence in Atlanta, Georgia and population characteristics associated with case concentrations. J Urban Health.

[20] Chen J, Chang H, Hammer M, D'Anna N, Gelberg K (2018). Using scan statistic to detect heroin overdose clusters with hospital emergency room visit data. OJPHI.

[21] Marks C, Carrasco-Escobar G, Carrasco-Hernández R, Johnson D, Ciccarone D, Strathdee SA, Smith D, Bórquez A (2021). Methodological approaches for the prediction of opioid use-related epidemics in the United States: a narrative review and cross-disciplinary call to action. Transl Res.

[22] Greene SK, Peterson ER, Balan D, Jones L, Culp GM, Fine AD, Kulldorff M (2021). Detecting COVID-19 clusters at high spatiotemporal resolution, New York City, New York, USA, June-July 2020. Emerg Infect Dis.

[23] Desjardins M, Hohl A, Delmelle E (2020). Rapid surveillance of COVID-19 in the United States using a prospective space-time scan statistic: detecting and evaluating emerging clusters. Appl Geogr.

[24] Alves H, Fernandes FA, Lima KPD, Batista BDDO, Fernandes TJ (2021). Incidence and lethality of COVID-19 clusters in Brazil via circular scan method. Rev Bras Biom.

[25] AlQadi H, Bani-Yaghoub M, Balakumar S, Wu S, Francisco A (2021). Assessment of retrospective COVID-19 spatial clusters with respect to demographic factors: case study of Kansas City, Missouri, United States. Int J Environ Res Public Health.

[26] Levin-Rector A, Kulldorff M, Peterson ER, Hostovich S, Greene SK (2024). Prospective spatiotemporal cluster detection using SaTScan: tutorial for designing and fine-tuning a system to detect reportable communicable disease outbreaks. JMIR Public Health Surveill.

[27] Martines MR, Ferreira RV, Toppa RH, Assunção LM, Desjardins MR, Delmelle EM (2021). Detecting space-time clusters of COVID-19 in Brazil: mortality, inequality, socioeconomic vulnerability, and the relative risk of the disease in Brazilian municipalities. J Geogr Syst.

[28] Aturinde A, Mansourian A (2022). Space-time surveillance of COVID-19 seasonal clusters: a case of Sweden. ISPRS Int J Geo-Inf.

[29] (2021). Update to the standardized surveillance case definition and national notification for 2019 novel coronavirus disease.

[30] Xu X, Wu Y, Kummer AG, Zhao Y, Hu Z, Wang Y, Liu H, Ajelli M, Yu H (2023). Assessing changes in incubation period, serial interval, and generation time of SARS-CoV-2 variants of concern: a systematic review and meta-analysis. BMC Med.

[31] Stephanie G (2016). Jaccard index / similarity coefficient. Statistics How To.

[32] Office for Human Research Protections (2020). OHRP guidelines on COVID-19. U.S. Department of Health and Human Services.

[33] 2018 Requirements (2018 Common Rule). U.S. Department of Health and Human Services.

[34] (2022). SaTScan User Guide for Version 10.1.

[35] O'Keefe KA, Shafir SC, Shoaf KI (2013). Local health department epidemiologic capacity: a stratified cross-sectional assessment describing the quantity, education, training, and perceived competencies of epidemiologic staff. Front Public Health.

[36] van den Wijngaard CC, van Asten L, van Pelt W, Doornbos G, Nagelkerke NJD, Donker GA, van der Hoek W, Koopmans MPG (2010). Syndromic surveillance for local outbreaks of lower-respiratory infections: would it work?. PLoS One.

[37] Xu F, Beard K (2021). A comparison of prospective space-time scan statistics and spatiotemporal event sequence based clustering for COVID-19 surveillance. PLoS One.

[38] Baker MG (2021). Occupational health surveillance as a tool for COVID-19 prevention. Am J Public Health.

[39] Baker MG, Peckham TK, Seixas NS (2020). Estimating the burden of United States workers exposed to infection or disease: a key factor in containing risk of COVID-19 infection. PLoS One.

[40] Luckhaupt SE, Horter L, Groenewold MR, de Perio MA, Robbins CL, Sweeney MH, Thomas I, Valencia D, Ingram A, Heinzerling A, Nguyen A, Townsend EB, Weber RC, Reichbind D, Dishman H, Kerins JL, Lendacki FR, Austin C, Dixon L, Spillman B, Simonson S, Tonzel J, Krueger A, Duwell M, Bachaus B, Rust B, Barrett C, Morrison B, Owers Bonner KA, Karlsson ND, Angelon-Gaetz K, McClure ES, Kline KE, Dangar D, Reed C, Karpowicz J, Anderson SM, Cantor S, Chaudhary I, Ellis EM, Taylor ML, Sedon A, Kocharian A, Morris C, Samson ME, Mangla AT (2023). COVID-19 outbreaks linked to workplaces, 23 US jurisdictions, August-October 2021. Public Health Rep.

[41] (2022). Labor & Industries Washington State.

[42] Pray IW, Grajewski B, Morris C, Modji K, DeJonge P, McCoy K, Tomasallo C, DeSalvo T, Westergaard P, Meiman J (2023). Measuring work-related risk of COVID-19: comparison of COVID-19 incidence by occupation and industry – Wisconsin, September 2020-May 2021. Clin Infect Dis.

[43] Brown CM, Vostok J, Johnson H, Burns M, Gharpure R, Sami S, Sabo RT, Hall N, Foreman A, Schubert PL, Gallagher GR, Fink T, Madoff LC, Gabriel SB, MacInnis B, Park DJ, Siddle KJ, Harik V, Arvidson D, Brock-Fisher T, Dunn M, Kearns A, Laney AS (2021). Outbreak of SARS-CoV-2 infections, including COVID-19 vaccine breakthrough infections, associated with large public gatherings—Barnstable County, Massachusetts, July 2021. MMWR Morb Mortal Wkly Rep.

[44] Inslee J (2021). Washington Ready.

[45] Inslee J (2021). Large event COVID-19 vaccine verification.

[46] (2021). Learn to Return Playbook.

[47] (2022). COVID-19 Outbreaks in Washington State K-12 Schools.

[48] Bonwitt J, Deya RW, Currie DW, Lipton B, Huntington-Frazier M, Sanford SJ, Pallickaparambil AJ, Hood J, Rao AK, Kelly-Reif K, Luckhaupt SE, Pogosjans S, Lindquist S, Duchin J, Kawakami V (2021). MMWR Morb Mortal Wkly Rep.

